# Phenotypic vulnerability of energy balance responses to sleep loss in healthy adults

**DOI:** 10.1038/srep14920

**Published:** 2015-10-08

**Authors:** Andrea M. Spaeth, David F. Dinges, Namni Goel

**Affiliations:** 1 Center for Sleep and Circadian Neurobiology, Perelman School of Medicine at the University of Pennsylvania, Philadelphia, PA; 2 Division of Sleep and Chronobiology, Department of Psychiatry, Perelman School of Medicine at the University of Pennsylvania, Philadelphia, PA

## Abstract

Short sleep duration is a risk factor for increased hunger and caloric intake, late-night eating, attenuated fat loss when dieting, and for weight gain and obesity. It is unknown whether altered energy-balance responses to sleep loss are stable (phenotypic) over time, and the extent to which individuals differ in vulnerability to such responses. Healthy adults experienced two laboratory exposures to sleep restriction separated by 60–2132 days. Caloric intake, meal timing and weight were objectively measured. Although there were substantial phenotypic differences among participants in weight gain, increased caloric intake, and late-night eating and fat intake, responses within participants showed stability across sleep restriction exposures. Weight change was consistent in both normal-weight and overweight adults. Weight change and increased caloric intake were more stable in men whereas late-night eating was consistent in both genders. This is the first evidence of phenotypic differential vulnerability and trait-like stability of energy balance responses to repeated sleep restriction, underscoring the need for biomarkers and countermeasures to predict and mitigate this vulnerability.

Epidemiological studies consistently find that short sleep duration (habitual sleep ≤6h/night) is a significant risk factor for weight gain and obesity^1^,^2^,^3^ and experimental studies have demonstrated that sleep restriction leads to increases in hunger^4^, impulsivity in response to food cues^5^, late-night eating^6^,^7^,^8^, portion sizes^9^, fat intake^8^,^10^, daily caloric intake^6^,^7^,^8^ and weight gain^6^,^7^ as well as attenuated fat-loss when dieting^11^. Furthermore, sleep loss disrupts metabolic hormones (e.g., ghrelin and insulin)^4^,^12^,^13^ and neural activity in limbic^14^, reward^15^ and salience^16^ networks which may underlie the observed changes in feeding behavior. In addition to these main effects, there are also group (gender and race) differences in food-intake^10^ and in weight-gain^6^ responses to sleep restriction, with men displaying a greater increase in caloric intake, late-night eating and weight gain than women. However, the extent to which individuals differ in phenotypic vulnerability to such responses is unknown.

Recent experimental studies have established that neurobehavioral responses (e.g., alertness, cognitive throughput, working memory, subjective sleepiness and mood) to sleep loss are phenotypic and stable within individuals^17^,^18^,^19^,^20^. There are large and reliable phenotypic differences in the degree of impairment caused by sleep loss such that some adults are highly vulnerable and show marked and increasing deficits during consecutive days of sleep restriction whereas other individuals are relatively resistant and show consistent neurobehavioral performance across protocol days; furthermore, individuals are consistent in their response to sleep loss when assessed multiple times^17^,^18^,^19^. It remains unknown whether an individual’s energy balance responses (caloric intake, late-night eating, and weight gain) to sleep loss are also phenotypic and trait-like over time and across exposures. These are critical and timely questions relevant to basic sleep and circadian rhythms research as well as patient care and clinical practice given marked population escalations in both the incidence of weight gain/obesity and curtailment of sleep^21^,^22^.

The current study assessed caloric intake, meal timing and weight change in order to examine how individuals responded to two chronic sleep restriction exposures separated by a long time interval. We hypothesized that similar to neurobehavioral responses, individuals would differ in their phenotypic vulnerability to the energy balance effects of sleep loss and that an individual’s energy balance responses to sleep loss would be stable and trait-like across time. Such findings would highlight the need for identifying biomarkers and countermeasures to predict and mitigate this vulnerability.

## Results

Healthy adults (n = 25) participated in two in-laboratory experiments, separated by at least 60 days, involving two nights of sufficient sleep followed by five nights of sleep restriction. Table 1 summarizes the demographic information for the study sample. During the week prior to study admittance for both sleep restriction exposures, participants were monitored in terms of their sleep patterns and weight. The change in weight during the week prior to study admittance (screening to admittance) did not differ between exposures (*P* = 0.92). Sleep duration and timing (assessed using wrist actigraphy) and chronotype (assessed using a morningness-eveningness questionnaire^23^) also did not differ during the week prior to sleep restriction exposures (*P*s > 0.10). There were no differences between men and women in age (exposure 1: p = 0.64, exposure 2: p = 0.68), BMI (exposure 1: p = 0.91, exposure 2: p = 0.91), or in the percentage of African Americans (p = 0.57).

**Figure 1:**
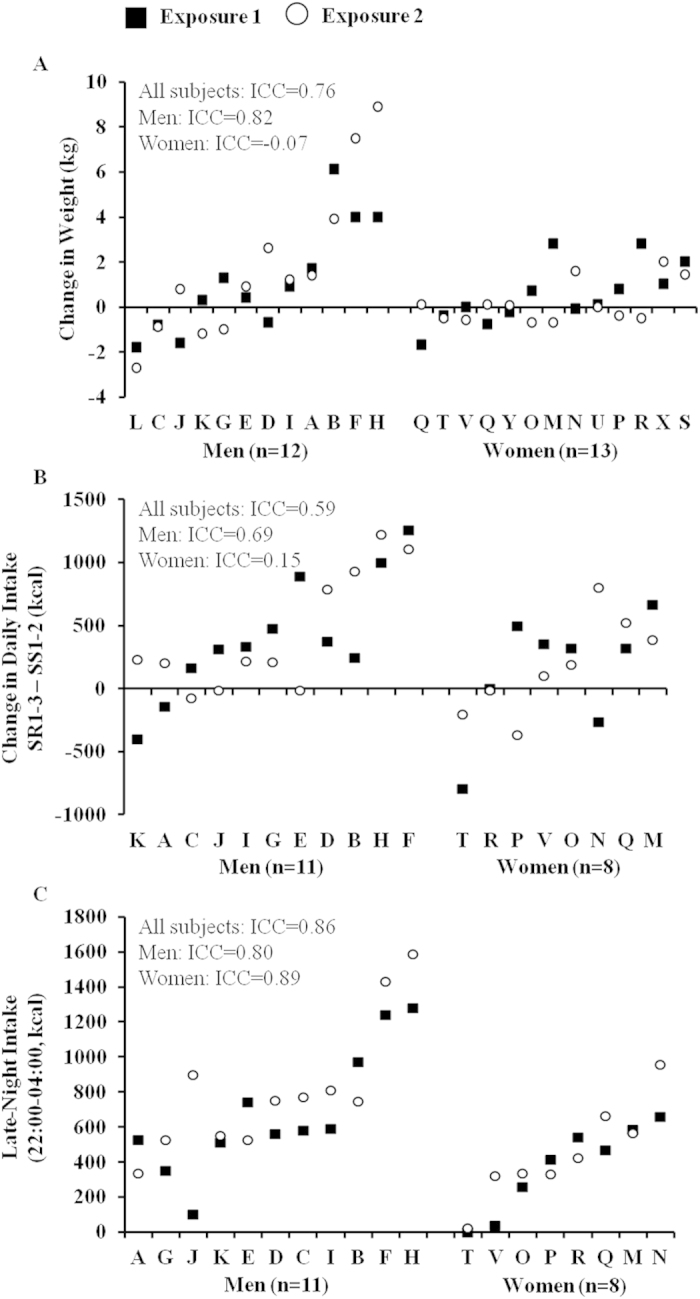
Individual differences and substantial phenotypic stability of energy balance measures to repeated sleep loss. (**A**) Change in weight (discharge minus admittance) during two separate laboratory sleep restriction exposures. (**B**) Daily caloric intake change (three days following sleep restriction [SR1-3] minus two days following sufficient sleep [SS1-2]) and (**C**) late-night intake (calories consumed from 22:00–04:00 during SR1-3) during two separate laboratory sleep restriction exposures. Participants (denoted individually with letters) are separated by gender and are plotted in ascending order based on mean weight-change/change-in-caloric-intake/late-night intake from both exposures. See text for ICC ranges.

During the study, there were large phenotypic individual differences in weight gain (study admittance to discharge) across participants (average change-in-weight during both exposures ranged from −2.3 to 6.5 kg). However, these in-study weight-change differences were consistent within individuals across exposures (*P* = 0.81), with a substantial intraclass correlation coefficient (ICC; 0.76, Fig. 1A). The weight-change ICC was nearly perfect for men (n = 12, ICC = 0.82) but not for women (n = 13, ICC = −0.07). Furthermore, when the sample was divided into groups based on BMI (using World Health Organization classification), both normal-weight (BMI < 25, n = 14) and overweight (BMI ≥ 25, n = 11) subjects exhibited substantial stability in the weight-change response to sleep loss (normal-weight ICC = 0.74, overweight ICC = 0.73).

In 19 participants, daily caloric intake was measured during both sleep restriction exposures. The change in daily caloric intake during sleep restriction (average intake during the first three days following sleep restriction [SR1-3] minus average intake during the two days following sufficient sleep [SS1-2]) showed large phenotypic individual differences across participants (average daily intake-change during both exposures ranged from −500.7 to 1178.2 kcal). However, individuals exhibited consistent caloric intake changes during sleep restriction across exposures (*P* = 0.77). These results showed a substantial ICC (0.59, Fig. 1B), which was due primarily to men (n = 11, ICC = 0.69) and not women (n = 8, ICC = 0.15).

Late-night intake (calories consumed from 22:00–04:00 during SR1-3) also showed large phenotypic individual differences (average late-night intake during both exposures ranged from 11.9 to 1434.1 kcal) that were highly reliable within participants across exposures as evident by an almost perfect ICC (0.86, Fig. 1C). Unlike daily intake, late-night intake ICCs were almost perfect for both men (0.80) and women (0.89). Similarly, late-night macronutrient composition (% of late-night calories derived from protein, carbohydrate and fat) was stable within participants across exposures (Ps > 0.59). Of significance, there was a substantial ICC for late-night fat intake (0.75) but not for late-night protein (ICC = 0.29) or carbohydrate intake (ICC = 0.39).

Notably, individuals exhibited consistency across energy balance responses when averaging both exposures: increased caloric intake during sleep restriction was significantly associated with greater late-night intake (*ρ* = 0.69, *P* = 0.001) and weight gain (*ρ* = 0.54, *P* = 0.017), and greater late-night intake was significantly associated with greater weight gain (*ρ* = 0.52, *P* = 0.023).

## Discussion

This is the first evidence of substantial phenotypic inter-individual differences and intra-individual stability in energy balance responses to sleep restriction, particularly in men. Men who gained a substantial amount of weight and increased their caloric intake to a significant degree during sleep restriction did so consistently during both exposures, suggesting they may be particularly vulnerable to the energy balance effects of sleep restriction. Conversely, men who lost or maintained weight during the study and did not show a substantial increase in caloric intake during sleep restriction also did so consistently during both exposures, suggesting they may be resistant to the energy balance effects of sleep restriction. Notably, hyperphagia and uncontrolled weight gain are associated with obesity, type II diabetes, hypertension and cardiovascular disease^24^. Therefore, men who are sleep deprived due to work obligations or lifestyle choices and who exhibit phenotypic vulnerability to the energy balance effects of sleep loss are at particularly heightened risk for developing these diseases.

We observed that both men and women exhibited marked phenotypic interindividual differences but intraindividual stability in late-night eating in response to sleep restriction. Animal studies have consistently shown that the consumption of calories during inappropriate times-of-day leads to greater weight gain than the consumption of the same amount of calories during the appropriate time-of-day^25^. Recent studies in humans have also highlighted the importance of meal timing for weight maintenance and health^26^,^27^,^28^. Adults who are particularly vulnerable to this sleep restriction response and who are awake during late-night hours due to shift work or sleep loss may be at heightened risk for weight gain, obesity and associated diseases.

The presence of stable individual differences in these energy balance responses to sleep restriction indicate these responses are phenotypic, with behavioral, physiological and genetic differences underlying these responses. Identification of biomarkers^29^ to predict an individual’s vulnerability to overeating and gaining weight when sleep deprived is a critical next step. Such possible biomarkers include genetic polymorphisms related to orexin signaling^30^, which have been implicated in both sleep-wake and feeding behaviors, and CLOCK genetic variants, which have been demonstrated to modify the relationship between sleep duration and BMI in a large population study^31^. In addition to genetic variants, individual differences in the response to peripheral physiological controls of eating from sleep loss may also play a role; examples include gastric emptying rates, insulin sensitivity, and ghrelin and leptin secretion^32^. Finally, eating behaviors and attitudes about food are also important to consider; indeed, the degree of disinhibited eating in adults may be particularly important^33^. Future studies are needed to systematically examine which behavioral and physiological factors predict the energy balance responses to sleep loss characterized in the current study.

The participants in this sample were healthy, between the ages of 22–50y, and had BMIs in the normal to overweight range. Notably, we observed stability in the weight-change response to sleep loss in both normal-weight and overweight adults. Our results may therefore not generalize to other groups, including obese individuals, adolescents or the elderly. Future research with larger sample sizes is needed to examine the stability of weight-change, caloric intake, meal timing, and energy expenditure responses to sleep loss in normal, overweight and obese individuals of varying ages.

Our findings highlight the importance of obtaining sufficient sleep for weight maintenance, particularly for certain individuals who are more vulnerable to the energy balance effects of sleep loss. They are of noteworthy and timely importance given marked societal escalations in weight gain, obesity and repeated, chronic sleep loss.

## Methods

### Participants

Healthy individuals, aged 22–50y, were recruited in response to study advertisements. Enrolled participants reported habitual nightly sleep durations between 6.5h and 8.5h, habitual bedtimes between 22:00 and 00:00, and habitual morning awakenings between 06:00 and 09:00. They had no evidence of habitual napping, no sleep disturbances (i.e., no complaints of insomnia, daytime sleepiness, or other sleep-wake disturbances), and an absence of extreme morningness or extreme eveningness, as assessed by questionnaire^23^. They were free of acute and chronic medical and psychological conditions, as established by interviews, clinical history, questionnaires, physical examinations, and blood (including a fasting blood glucose test) and urine tests, and were not taking any regular medications (except oral contraceptives). Participants were monitored at home with actigraphy, sleep-wake diaries, and time-stamped call-ins to assess bedtime and wake time during the week prior to the in-laboratory phase and the week after the laboratory phase for both exposures. They were nonsmokers and had a BMI ranging between 19–30 kg/m^2^. They did not participate in shift work, transmeridian travel, or irregular sleep/wake routines in the 60 days prior to the study. Sleep disorders were excluded by a night of laboratory polysomnography and oximetry measurements. Participants were not permitted to use caffeine, alcohol, or tobacco in the week before the laboratory experiment, as verified by blood and urine screenings.

Twenty-five healthy adults (age at first study: 33.3 ± 8.8y; 12 men and 13 women [Table 1], 76% African American, 20% Caucasian, 4% Asian) participated in two laboratory studies separated by ≥60 days (range = 60–2132 days, median = 354 days, mean = 621 days). Protocols were approved by University of Pennsylvania’s Institutional Review Board. Participants provided written informed consent, which was in accordance with the Declaration of Helsinki, and received compensation.

### Procedure

Participants were enrolled in two laboratory studies involving sleep restriction (5 consecutive nights of 4 h time-in-bed [TIB]/night, 03:00–07:00 or 04:00–08:00). At least two nights of sufficient sleep (SS1-2; 8–12 h sleep/night, 21:00–07:00/22:00–08:00/22:00–10:00) preceded nights of sleep restriction during both exposures. Both studies were conducted in the Sleep and Chronobiology Laboratory at the Hospital of the University of Pennsylvania. During both studies, participants were monitored continuously with daily clinical checks of vital signs and symptoms by nurses (with an independent physician on call) and were not permitted to leave the laboratory. Participants were ambulatory and were allowed to watch television, read, play video or board games, and perform other sedentary activities between test bouts (which were completed while sitting at a computer) but were not allowed to exercise. Light levels in the laboratory were held constant at <50 lux during scheduled wakefulness and <1 lux during scheduled sleep periods. Ambient temperature was maintained between 22°–24 °C. Participants were behaviorally monitored by trained staff continuously throughout the protocol to ensure adherence.

### Measures

Weight was assessed by nurses in the Clinical and Translational Research Center at the Hospital of the University of Pennsylvania at screening, admittance and discharge.

Participants selected their meals and snacks by choosing from various menu options, by selecting additional food and drink available in the kitchen within the laboratory suite (which included a refrigerator, microwave, and toaster oven) and by making requests to the study staff. All food was weighed and recorded prior to being provided to the participants. To enhance the measurement accuracy of each food item’s weight, items were served in individual containers. Each day, a detailed description of the items and the amount consumed and intake time were recorded by trained monitors. Additionally, any food and drink that was left over after each meal was weighed and recorded. The intake data were entered into The Food Processor SQL program (ESHA Research, Salem, OR), a validated^34^ professional nutrition analysis software and database program that provides components of food and drink intake including calories and macronutrients.

### Statistical Analyses

Paired-t-tests (comparing outcome measures for both sleep restriction exposures) and intraclass correlation coefficients^35^ (ICC: two-way mixed, absolute agreement, average measures) assessed the interindividual differences and intraindividual stability of energy balance responses to sleep restriction (SPSS v20). The following established ranges characterize ICCs and reflect increasing stability of observed interindividual differences: 0.0–0.2 (slight); 0.2–0.4 (fair); 0.4–0.6 (moderate); 0.6–0.8 (substantial); and 0.8–1.0 (almost perfect)^35^. Spearman’s rho assessed the relative rank of individuals across energy balance measures.

## Additional Information

**How to cite this article**: Spaeth, A. M. *et al.* Phenotypic vulnerability of energy balance responses to sleep loss in healthy adults. *Sci. Rep.* **5**, 14920; doi: 10.1038/srep14920 (2015).

## Figures and Tables

**Table 1:** Study Sample Characteristics.

Subject	Age		BMI (kg/m^2^)		Days Between Studies
	Exposure 1	Exposure 2	Exposure 1	Exposure 2	
*Men*					
A	22	22	21.8	21.9	111
B	49	50	20.9	21.8	160
C	25	26	27.7	28.7	179
D	46	46	21.6	21.0	251
E	27	28	22.1	21.7	270
F	37	38	19.3	20.4	341
G	43	44	23.9	24.4	398
H	30	32	21.4	19.1	559
I	40	42	27.1	27.7	792
J	47	50	20.1	21.4	890
K	28	31	29.3	29.7	956
L	26	29	28.0	29.4	1159
*Women*					
M	25	25	21.6	21.6	60
N	31	32	21.6	20.7	163
O	42	42	24.6	23.5	201
P	32	33	28.2	29.1	235
Q	25	26	20.9	19.5	250
R	41	42	25.8	26.6	323
S	30	31	25.2	23.6	354
T	22	23	24.0	25.0	375
U	42	45	22.9	21.1	1025
V	33	37	24.5	30.3	1328
W	24	28	20.5	20.9	1338
X	22	27	24.2	25.4	1676
Y	43	49	24.6	26.1	2132
